# Integrating PrEP into HIV care clinics could improve partner testing services and reinforce mutual support among couples: provider views from a PrEP implementation project in Kenya

**DOI:** 10.1002/jia2.25303

**Published:** 2019-07-19

**Authors:** Josephine B Odoyo, Jennifer F Morton, Kenneth Ngure, Gabrielle O'Malley, Kenneth K Mugwanya, Elizabeth Irungu, Merceline Awuor, Annabell Dolla, Fernandos Ongolly, Elizabeth A Bukusi, Nelly R Mugo, Jared M Baeten, for the Partners Scale‐Up Project Team

**Affiliations:** ^1^ Kenya Medical Research Institute Center for Microbiology Research Nairobi Kenya; ^2^ Department of Global Health University of Washington Seattle WA USA; ^3^ Department of Community Health Jomo Kenyatta University of Agriculture and Technology Nairobi Kenya; ^4^ Kenya Medical Research Institute Center for Clinical Research Nairobi Kenya; ^5^ Department of Obstetrics and Gynecology University of Washington Seattle WA USA; ^6^ Department of Medicine University of Washington Seattle WA USA; ^7^ Department of Epidemiology University of Washington Seattle WA USA

**Keywords:** HIV, partner notification services, disclosure, ART, PrEP, HIV care continuum

## Abstract

**Introduction:**

Partner notification services (PNS) increase the HIV status knowledge and linkage to care and treatment. However, it is unclear if PNS can facilitate linkage of HIV‐negative partners to prevention services such as pre‐exposure prophylaxis (PrEP). Using qualitative methods, we explored provider perspective regarding the interaction of PrEP availability, PNS and antiretroviral treatment (ART) outcomes within a project integrating PrEP services into HIV care clinics in eight counties in western and central Kenya.

**Methods:**

From May 2017 to August 2018, data on integrated PrEP service delivery including its interaction with PNS were collected through 71 key informant in‐depth interviews with healthcare providers and 24 standardized technical assistance reports summarizing implementation at the participating clinics. Thus, the perspective was from that of providers; analyses focused on emergent themes relating PNS to PrEP and ART services.

**Results:**

Providers found that PrEP integration provided an additional concrete prevention option for HIV‐negative partners and created a motivation to offer PNS to persons living with HIV. PrEP availability also seemed to operate as an incentive for those living with HIV to participate in PNS, which in turn enhanced identification of potential PrEP clients and created an environment for discussing HIV transmission risk. Providers commented that initiating HIV‐negative partners on PrEP enhanced mutual monitoring of health outcomes, including improved adherence to ART by partners living with HIV. Clinics noted prioritizing people living with HIV with detectable viral loads for PNS in order to identify HIV‐negative partners who would benefit most from PrEP. Providers felt motivated by the apparent synergistic interaction of PNS, PrEP and ART.

**Conclusions:**

Providers felt that the integration of PrEP into HIV care clinics stimulated the provision of PNS, and PNS was used to improve the identification of potential PrEP clients. The integrated combination of PNS, PrEP and ART is synergistic and should be promoted in HIV clinics.

## 1 Introduction

Antiretroviral treatment (ART) to reduce the infectiousness of persons living with HIV and pre‐exposure prophylaxis (PrEP) for persons at‐risk of HIV to prevent HIV acquisition are extraordinarily effective strategies for achieving HIV epidemic control worldwide 1, 2, 3, 4. For both PrEP and ART, knowledge of one's own HIV status is essential. In addition, knowing the HIV status of one's partner(s) might effectively facilitate access, uptake and adherence to ART and PrEP. Assisted HIV partner notification services (PNS) – a strategy under which providers offer HIV testing to sexual partners of consenting people living with HIV (PLHIV) – increases the fraction of partners tested and is recommended by the World Health Organization 5, 6, 7, 8. While PNS has been shown to improve the diagnosis of partners living with HIV, whether PNS can facilitate linkage to prevention services (like PrEP) is unknown. In addition, social support, which results from PNS, can enhance mutual emotional resilience within partnerships and ultimately improve treatment adherence 9, 10.

HIV care clinics offer an existing platform to integrate the delivery of PrEP and ART. We previously reported, in a demonstration project at four clinics in Kenya and Uganda, that integrated offering of PrEP and ART to HIV serodiscordant couples resulted in near‐elimination of HIV transmission risk in that population 1, 3. A national scale‐up of PrEP delivery for HIV serodiscordant couples in Kenya (the Partners Scale‐up Project) is currently ongoing, with the goal of catalysing nationwide integration of PrEP in HIV care clinics using existing facility infrastructure and personnel capacity 11. Concurrent with Kenyan national scale‐up of PrEP, the Kenyan Ministry of Health recently issued guidance recommending PNS to increase the uptake of HIV testing in partners of PLHIV, creating an enabling environment for PNS to interface with other services 12. We used provider qualitative interviews in the ongoing Partners Scale‐Up Project to assess the impact, from the perspective of providers, of PrEP availability on partner testing and potential resultant synergy among PNS, PrEP and ART services.

## 2 Methods

### 2.1 Study design

**Figure 1:**
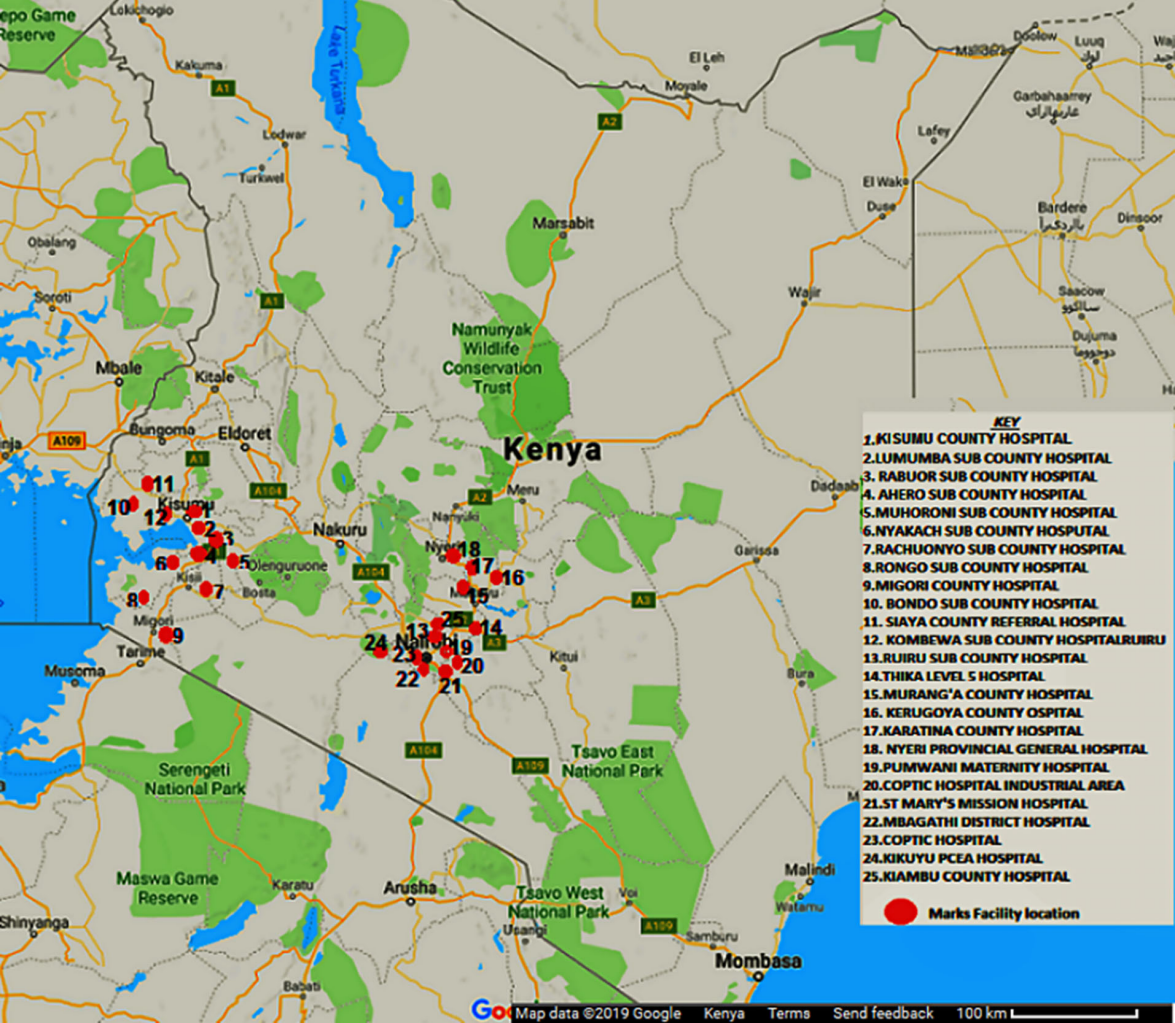
Map showing Partners Scale‐Up Project clinic locations.

The Partners Scale‐Up Project is an implementation study documenting the process of catalysing PrEP delivery in Kenya (Clinicaltrials.gov NCT03052010) 11. The work is being conducted in 24 public HIV care health facilities in central and western Kenya, specifically Kiambu, Kirinyaga, Muranga and Nyeri counties (HIV prevalence approximately 4%) and Homabay, Kisumu, Migori and Siaya counties (HIV prevalence approximately 15%) (Figure 1). All clinics are Level 4 or 5 health facilities with a minimum of 3500 patients on ART; most are staffed by Kenya Ministry of Health employees, a minority are supported by missionary/religious institutions. The study facilities were selected because of administrator and county government support of PrEP integration, availability of healthcare staff for training about PrEP delivery, and agreement to participate in ongoing research documentation of the PrEP integration and delivery process.

Between January and July 2017, providers in these clinics were trained in PrEP delivery, using training manuals covering PrEP and ART delivery developed in part with the Kenya Ministry of Health 11. As of August 2018, over 3500 individuals had initiated PrEP in these facilities, most (87%) in HIV serodiscordant relationships 13. The Partners Scale‐Up Project does not employ or pay staff at facilities; instead, project staffs are engaged in ongoing technical assistance to clinics and conduct qualitative interviews with clinic providers to evaluate best practices of integrated PrEP and ART delivery. Technical assistants (TAs) were clinical experts in PrEP themselves (nurses, clinical officers, etc.) who had worked in both research and clinical care delivery and who trained clinic staff and provided periodic onsite assistance. Qualitative interviewers were separate staff from those conducing TA visits. The project was reviewed and approved by the Institutional Review Boards of the University of Washington and the Kenya Medical Research Institute; written consent was obtained for qualitative interviews.

### 2.2 Data collection

Two data sources contribute to the present analysis: key informant interviews with health providers and clinic technical assistance reports. Data were collected between May 2017 and August 2018. For interviews, semi‐structured interview guides included probes about how PrEP delivery was integrated into clinic practices, how information about PrEP was disseminated to persons living with HIV attending the clinic and more broadly, and the provider experiences in delivering PrEP. All providers who were interviewed were either directly involved in PrEP delivery or responsible for overseeing PrEP delivery. Healthcare providers were purposively sampled; members of the TA team recommended providers to be interviewed based on their support of or hindrance to PrEP delivery. Each participating provider was interviewed for one to two hours in English or Kiswahili based on personal language preference. Interviews were audio‐recorded and transcribed (and translated if necessary); accuracy of the transcripts was verified by at least one other research team member who had not participated in the transcription process.

Information on facilitators and barriers to PrEP delivery was collected and documented by TA visits to clinics, which occurred approximately once a month for two to eight hours. TA observations and conversations with clinic staff were documented in TA reports generated after each visit using structured guides; comprehensive TA summaries were periodically compiled across reports. TA reports described successes and challenges regarding demand creation, identification of PrEP clients, PrEP delivery, workforce, commodities and retention. Between monthly visits, TA teams also documented phone calls they received from clinic staff, which occurred occasionally, including reason for the calls and their resolution. Qualitative interviewers and TAs held weekly meetings to discuss concerns and gaps in knowledge or delivery and to ensure that the two data collection systems were aligning 11.

### 2.3 Data analysis

Qualitative interview transcripts were coded and analysed using Dedoose (https://www.dedoose.com/, version 8.0.42); coding and analysis were conducted using a thematic approach, which allows for a more flexible form of analysis and examination of the participants’ perspectives and a more comprehensive account of the data 14. Codes were applied to all the transcripts to derive the descriptive and inductive thematic analysis of data gathered during the study. Transcripts were coded by two experienced social scientists who read transcripts as they became available and inductively listed main codes and fine codes as they emerged to develop a codebook. They then compared the two codebooks and discussed and reached consensus where there were discrepancies to come up with a final codebook that was used in the coding. Similarly, the two social scientists double‐coded all the transcripts and compared the resultant coded transcripts and for inter‐coder agreement. Where inter‐coder agreement was not achieved, they held discussion meetings in the presence of the lead author to collectively agree on emergent themes [15,16]. We merged common codes into defined themes and clusters that informed our conclusions.

The domains defined in the Consolidated Framework for Implementation Research (CFIR) guided development and analysis of the TA reports. Descriptive information characterized facility‐level innovations that improved PrEP uptake process and what went well, what was challenging and why, and options and opportunities for further improvement. Themes related to PrEP demand creation and identifying PrEP users were entered into a matrix in Microsoft Excel and analysed guided by the rapid and rigorous qualitative data analysis (RADaR) approach 17.

## 3 Results

Key informant interviews were conducted with 71 health providers (Table 1). TA reports were reviewed from all 24 clinics. Across the qualitative data and TA reports, the interaction between PNS, mutual support, and PrEP and ART services emerged as an important theme.

**Table 1:** Providers participating in key informant qualitative interviews

Cadre	Total n = 71
Doctors	0 (0%)
Nurse counsellors	4 (6%)
Clinical officers	22 (31%)
HIV testing counsellors	21 (30%)
Adherence counsellors	13 (18%)
Health records	3 (6%)
Social worker	5 (7%)
Peer educator	2 (3%)
Pharmaceutical technologists	1 (1%)
Characteristics	
Age, median (range)	32 (23 to 65)
Months of experience, median (range)	48 (3 to 204)
Gender	56% female

### 3.1 PNS context and implementation

Kenya adopted PNS as a strategy to identify persons living with HIV and link them to care and treatment 18. For the clinics in this study, a variety of PNS options were noted: the most common was encouraging persons living with HIV to bring their partner for HIV testing services, often with a referral invitation card. One clinic reported making phone calls to encourage partners testing, and six clinics sent an outreach worker. In contrast, some clinics did much less with PNS, with TA reports documenting that two rarely offered PNS services and five others did so inconsistently; three clinics did not conduct PNS until PrEP roll out. Approximately one‐quarter of clinics only conducted PNS for clients once – at the time of initial HIV diagnosis; the rest; however, considered PNS as an ongoing process with periodic queries about whether partners had been recently tested and if there were any new sexual partners. This kind of chart review occurred every three months for some clinics, six months for others, and in a more targeted fashion in the remaining (e.g. only for known HIV serodiscordant couples or only for persons living with HIV with detectable HIV plasma viral loads).

### 3.2 PNS as a tool for providers in integrated PrEP delivery

Providers noted that the introduction of PrEP into HIV care offered an important new service for HIV serodiscordant couples. The clinics used a number of strategies to identify ideal candidates for PrEP, including conducting health talks to the clients in the waiting bay, displaying PrEP posters, distributing brochures, hosting support groups for HIV serodiscordant couples, and identifying PLHIV enrolled in the clinic with high viral loads (who would specifically be queried about partners). Discussions of PrEP in the media and radio talk shows were also cited by providers as strategies for increasing PrEP awareness. Several providers reported that PNS was among the most successful strategies they used for identifying PrEP clients:

“Always if somebody comes alone for testing and they are asked if they have a family, now with the partner notification system they are encouraged to come along with their partners so the family status is known not you as an individual. Yeah so if you are positive and your partner is negative then of course you are encouraged to take PrEP… I think I can attribute that to the PNS thing that the HTS are embracing nowadays….” (Female Nurse, Central).

“One, for the clients who are HIV positive, we invite their partners through doing family and partner testing whether at home or through calling and then [when] they come we test them. If they are negative we recruit them [to PrEP]. Secondly when we go through their files, like the ones I have here, we are able to find their contacts of these partners and recruit them. Thirdly through the use of discordant couple register which is well updated, we are able to find those who are eligible [for PNS and for PrEP]” (Male Clinical Officer, Western).

With the introduction of PrEP, clinics reported that HIV counsellors were more consistent with, and more enthusiastic about, talking with newly‐diagnosed PLHIV about PNS as a means of identifying HIV‐negative partners, since PrEP offered a concrete prevention strategy. Prior to the availability of PrEP, they reported that they would only identify those who are positive and link them to treatment, but they felt they had “nothing to offer” to the negative partner; thus, in their view, the availability of PrEP enhanced PNS services. Other providers spoke of PNS and PrEP as mutually reinforcing – that is, the availability of PrEP motivated providers to offer PNS and knowledge of benefits of PrEP motivated the partners to take up PNS and in turn get linked to PrEP if HIV negative. Finally, providers also spoke of how PrEP and PNS interfaced with ART services, with some clinics periodically reviewing charts of patients on ART to identify those with high viral loads for targeted PNS:

“During [multi‐disciplinary team] meetings we discuss high viral load clients, if we find one that the partner's status is not known or is (HIV) negative we follow them at home to test the partner or call the clients to come [to the clinic] with the partner for testing and if negative we tell them about PrEP and enroll those willing on PrEP” (Female Clinical Officer, Western).

TA reports and interviews documented that health talks, which were routinely given in the mornings at the waiting bay as clients who are scheduled for the day wait to be attended, incorporated PrEP messaging, providing PLHIV with knowledge about the benefits of PrEP and potentially motivating them to bring their partners to the clinic.

### 3.3 PNS, with PrEP, as a tool for mutual support and sustained serodiscordant relationships

Before the introduction of PrEP into HIV care centres, providers commonly reported that many PLHIV struggled with disclosing their status to their partners for fear of relationship dissolution. However, with PNS services that incorporated PrEP, providers reported they could more confidently encourage PNS and it was their impression that persons living with HIV seemed more willing to provide their partner's contact details so HIV counsellors could call them and encourage them for testing and PrEP initiation:

“That one [i.e., PNS with PrEP as a component] is done so much with our clinical team and our social worker. From the team, we are able to get the discordant couples and we are able to talk on how we can help them. In case we get a client that tells you that I have a partner but doesn't know my status and what…we take up issues of disclosure. Now we take that client as a special client in a way. On the other side he is a special client because of the high viral load and on the other side he is a special client because the partner might end up being a PrEP client” (Female Counselor, Western).

The process of PNS at some point ultimately requires disclosure of HIV status by persons living with HIV, and providers reported that PrEP availability in clinics made it easier to disclose HIV status to partners:

“The coming of PrEP has improved disclosure because now clients feel that even if they disclose life will continue as normal, and we even have two adolescents who have come with their partners who are negative and we have offered them PrEP here. And that way…previously they had not disclosed, but when they came with these partners that just recently diagnosed and now they were coming for PrEP, so it has some evidence that it has improved disclosure” (Male Clinic In‐Charge, Central).

In addition, providers reported that the introduction of PrEP into HIV care centres offered an avenue for HIV serodiscordant couples to engage in HIV prevention as a couple, with each member of the couple provided a prevention option: ART for the partner living with HIV and PrEP for the HIV‐negative partner. Service providers reported appreciating that PrEP provides additional motivation for couples to attend the clinic together, which in turn encouraged providers because of the benefits they saw in serving couples rather than individuals. Furthermore, PNS increased uptake of couples’ engagement in facility events that target serodiscordant couples, like support groups.

Moreover, providers reported that they were able to give information together and hence they would discuss HIV‐related issues as a couple, such as how to cope with challenges of serodiscordance; providers also reported that they felt such joint sessions improved communication and adherence support:

“I usually find it, it is very much easier to enroll clients when they come as a couple because they can now understand the…maybe the source of infection and also you can also be able to tell them in case of anything like discordance, you can counsel them when they are together. And again it will assist in disclosure. Sometimes when you test one of the clients when the other one is not there, it will be difficult for the one to disclose to the partner. So usually I find it very much easier when they are together in the facility unlike when they are single clients” (Female Clinical Officer, Western).

### 3.4 Providers felt motivated when they observed and saw synergistic interactions of PNS, PrEP and ART

TA reports repeatedly emphasized that the joint availability of PrEP and PNS seemed to motivate providers in ART clinics, for increased identification of HIV‐negative partners who would benefit from other HIV prevention services (including but not just PrEP). Importantly, enrolment of HIV‐negative partners in PrEP, as a result of PNS, was reported by providers as enhancing mutual monitoring of health outcomes, particularly adherence to ART and was particularly motivating for providers, whose primary activity was achieving HIV treatment success at their clinic. For example, HIV‐negative persons on PrEP inquired about their partners’ latest viral load result when couples visited the clinic for ongoing care; many partners knew that PrEP delivery was linked to viral suppression, with Kenyan guidelines recommending PrEP discontinuation when viral suppression was achieved. Providers became more aggressive in identifying ART treatment failure, and, because of PrEP, PLHIV were motivated to disclose their HIV status and could thus be more adherent (because they did not have to hide that they were taking medication):

“One we have discordant couple psychosocial support group and during these psychosocial support groups we advocate them to come with their partners and for testing and even their children and they have found the support group to be very important and they do keep their appointment and they are taking their ART well” (Male Clinical Officer, Western).

“The issue of disclosure was a very big issue in the past, however, due to the introduction of PrEP lack of disclosure has been eliminated because now the fear that what if now I get tested…, what if now I let my partner know my status and yet we are of different status…? So the fact that PrEP is on board, disclosure has been a key issue and we all know that when disclosure is done and the right partner in the right way, adherence is enhanced” (Female Adherence Counselor, Western).

“When these clients are initially diagnosed with HIV they have several questions and anxieties, when you introduce the issue of PrEP to the client they have reliefs, … when you advise them that they have to take their drugs at the same time it improves adherence and they have good relationships…now they feel that they are enjoying sex life so that has improved” (Female Clinical Officer, Central).

### 3.5 Challenges of PNS in the context of PrEP and ART

Although PNS was often successful, clinic staff also reported challenges. When persons living with HIV chose to bring their partners for testing, doing so could take months, during which time there was ongoing transmission risk. In addition, sometimes the client living with HIV did not accompany the partner for testing, making it hard for the HIV counsellor to introduce couples‐based prevention approaches, which were felt to be the ideal. Some PrEP clients were uncomfortable attending HIV clinic (even for PNS, or later for ongoing PrEP engagement), which providers interpreted as in part being related to fear of being branded as persons living with HIV. Some clinics worked through these challenges by expanding PrEP services to other departments outside of HIV care clinics (e.g. to maternal‐child health departments in the same facility).

## 4 Discussion

**Figure 2:**
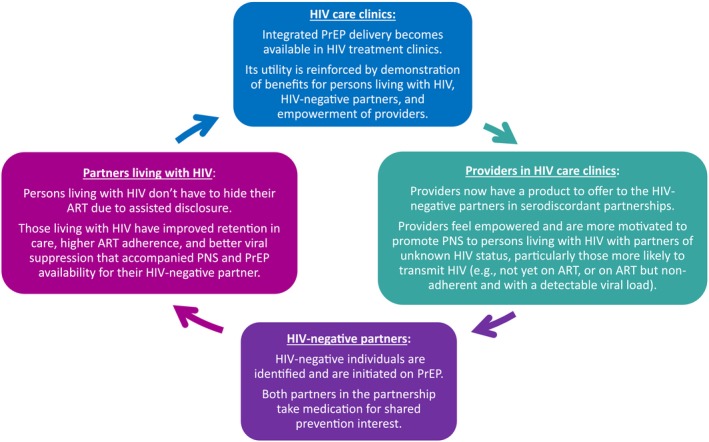
Conceptual framework relating the integration of PrEP into HIV care clinics on PNS and HIV treatment outcomes, as informed by the qualitative data, from the provider perspective, included in this study.

In this ongoing project integrating PrEP into selected HIV care clinics in Kenya, we used qualitative and implementation science methods to explore provider perspectives and practices related to the interface of PNS, PrEP and ART services. We found that PNS served as an entry point to PrEP services and the availability of PrEP services was reported to make PNS more appealing, both to providers and, from the provider perspective, to persons living with HIV. In addition, providing PNS and PrEP services together seemed to reduce barriers (including stigma and disclosure) to ART adherence for those living with HIV, which further reinforced the benefits of integrated PNS and PrEP for HIV care clinic staff. Together, these results suggest potentially important synergies between PNS, PrEP and ART that are positively reinforcing for both providers and their relationship with their clients (Figure 2).

Kenya offers a strong enabling environment for PNS, PrEP and ART provision. HIV testing services at scale have been a part of Kenya's HIV control programme for many years 19, and recent guidelines support PNS, PrEP for all at‐risk persons, and ART regardless of CD4 count 18, 20. Key work demonstrating the impact of PNS was done in Kenya, showing that PNS can be an effective approach to increase partner testing, particularly for identifying PLHIV and linking them to care and treatment 5, 6. Our findings extend that work to linkage of HIV‐negative partners to PrEP.

Notably, PNS, along with PrEP, helps to engage clients in HIV care to discuss health issues as a couple. Other work has suggested that delivering both ART and PrEP together in a clinical setting could motivate ART initiation [3,21]. We found that providers reported that the availability and use of PrEP by partners appeared to help ART adherence in some persons living with HIV who had previously been non‐adherent. PNS and PrEP together motivated providers to offer services for couples rather than individuals, including the opportunity for both partners to know their HIV status together. Mutual support in treatment decision‐making can improve clinic attendance and adherence [22,23]. We found that providers repeatedly brought up anecdotal cases in which the availability of PrEP encouraged persons living with HIV who had been poorly adherent to ART to bring in partners for testing and PrEP linkage and that in turn resulted in improved ART use as well.

Invitation letters were a common approach to identifying partners for testing and we found that clinics used those as well as other approaches such as health talks, phone calls by providers, and serodiscordant couples’ activities to increase partner testing and sustain linkage to care. More work to understand the benefits of different approaches to PNS, specifically as related to engagement to PrEP, is needed. Public HIV care clinics, like those participating in the present evaluation, offer an ideal setting to offer PNS, PrEP and ART under one roof, with HIV testing, counselling, and care expertise. As countries, like Kenya, expand both PrEP and PNS services, HIV testing services within or attached to HIV care clinics may be a robust starting point for discussions of partner testing and couples‐based prevention.

Despite the observed successes of PNS, the study highlighted challenges, consistent with other studies. The barriers to uptake of partner testing have included mistrust of public health workers, stigma and shame of knowing that they have been diagnosed with HIV and fear of notifying their partners 24, 25, 26. The study further showed that some partners expressed concerns about confidentiality and stigma.

The diverse settings in this study brought out a range of health provider perspective of PrEP service provision that may give a reasonable representation of provider opinions in Kenya in general. One of the limitations of this study is that we are unable to precisely give the numbers of those who may have reported resistance to partner testing especially at the community level since this study was facility‐based and our methodology was largely qualitative. Our interviews focused on health care providers’ perspectives in describing PNS; a deeper understanding of clients’ perspective is needed as well. Finally, we do not present quantitative data to know the relative increase of PNS offering or uptake after versus before PrEP integration.

## 5 Conclusions

PNS is an innovative approach that broadens avenues for discussions by couples and a window to increase partner testing. We found that, from the provider perspective, PrEP appears to interface positively with PNS, with PNS offering an entry point for PrEP uptake and PrEP creating an incentive for PNS. The integrated combination of PNS and PrEP services, embedded into existing ART programmes, could result in synergies that enhance uptake and utilization of partner testing, PrEP and effective HIV treatment.

## Competing interest

The authors declare that they have no competing interests.

## Authors’ contributions

J.B.O., K.K.M. and J.M.B. contributed to conceptualizing and designing the study. M.A., A.D., F.O., J.F.M. and E.O. acquired the data. J.B.O., J.F.M. and K.N. analysed the data. J.B.O., J.F.M. and J.M.B. wrote the paper. All authors reviewed and edited the manuscript and approved the final version.
